# Severe acute kidney injury associated with progression of chronic kidney disease after critical COVID-19

**DOI:** 10.1186/s13054-021-03461-4

**Published:** 2021-01-25

**Authors:** Michael Hultström, Miklos Lipcsey, Ewa Wallin, Ing-Marie Larsson, Anders Larsson, Robert Frithiof

**Affiliations:** 1 Anesthesia and Intensive Care Medicine, Department of Surgical Sciences, Uppsala University Hospital, Uppsala University, Entrance 78, etg 4, 75185 Uppsala, Sweden; 2 Integrative Physiology, Department of Medical Cell Biology, Uppsala University, Uppsala, Sweden; 3 Hedenstierna Laboratory, CIRRUS, Anesthesiology and Intensive Care, Department of Surgical Sciences, Uppsala University, Uppsala, Sweden; 4 Clinical Chemistry, Department of Immunology, Genetics and Pathology, Uppsala University, Uppsala, Sweden

## Dear Editor,

Corona virus disease 2019 (COVID-19) is associated with acute kidney injury (AKI) in patients that require critical care [1]. As we have previously reported, the association between clinical AKI and biomarkers was not strong, perhaps indicating less renal tissue damage [2]. Further, the preceding illness with high fever and restrictive fluid treatment in intensive care (ICU) may cause AKI through dehydration [3]. The mechanisms of AKI in COVID-19 may include direct viral injury [4]. However, urinary viral load in this cohort was low even in patients with severe AKI [5]. This study reports the prevalence of chronic kidney failure (CKD) three to 6 months after discharge in a cohort COVID-19 ICU patients. A total of 122 patients were included during their ICU stay. We excluded 3 who did not have COVID-19, 33 who died, and 26 who declined follow-up or did not have creatinine analysis. This left 60 patients analysed in the present study.

Baseline creatinine and eGFR were extracted from the medical records from the preceding year and used for CKD definition. AKI during the ICU-stay was defined according to KDIGO-criteria [6]. Kidney function at follow-up was estimated from plasma creatinine.

Data are expressed as mean ± standard deviation, median (IQR) or number of patients (%). Statistical differences were tested using Student’s *t* test, Wilcoxon’s Rank Sum Test or Chi-square-test as appropriate. *p* < 0.05 was considered significant.

**Fig. 1:**
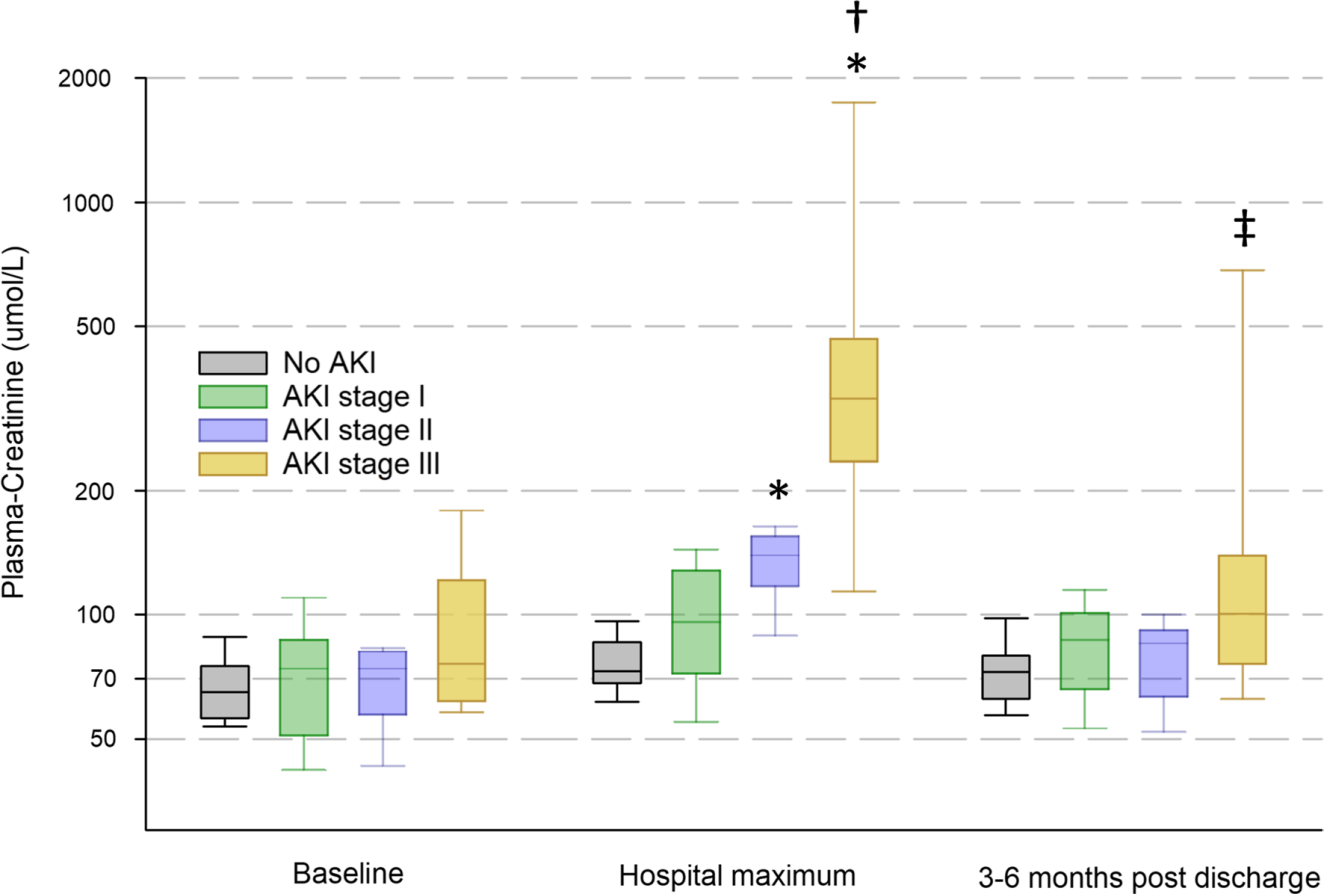
Progression of plasma creatinine in patients who developed different stages of AKI (No AKI *n* = 27, Stage 1 *n* = 19, Stage 2 *n* = 6, Stage 3 *n* = 8) during their ICU stay. A mixed model ANOVA on the logarithm of the concentration showed a significant difference in creatinine between stages (*p* = 3.5*10^–15^), as well as time-points (*p* = 1.4*10^–8^), with a highly significant interaction (*p* = 2.3*10^–9^). *Indicates significant difference compared to patients who did not develop AKI, ^†^Compared to baseline for the same stage, and ^‡^compared to hospital maximum for the same stage by TukeyHSD

Before COVID-19 onset only six patients had CKD stage 3 or higher (Table 1). During intensive care 27 (45%) patients did not develop AKI, 19 (31%) developed stage 1, 6 (10%) stage 2, and 8 (13%) stage 3. Patients had higher maximal creatinine (*p* = 3.5*10^–15^) based on the level of AKI. This was driven by a significant increase in creatinine from baseline (*p* = 1.4*10^–8^), that was expectedly more pronounced with increasing AKI stage (*p* = 2.3*10^–9^). The increase tended to normalise at follow-up, but creatinine remained higher in patients with stage 3 AKI than those who never developed AKI during the ICU-stay (Fig. 1).

**Table 1:** Patient characteristics of 60 followed-up after critial COVID-19 with kidney function three to six months after discharge. 50 patients (83%) did not progress, while 10 (17%) showed progression to a higher CKD stage. *p* indicates indicates probability of chance difference between patients without progression and patients with progression. Only severity of acute kidney injury (AKI) in the form of KDIGO stage 3 or dialysis, or duration of AKI in the form of acute kidney disease (AKD) were risk factors for CKD progression

	All patients	No progression	CKD progression	*p*
Demographics	n = 60	n = 50	n = 10	
Age	60 ± 12	60 ± 13	59 ± 10	NS
Women	17(28%)	15(30%)	2(20%)	NS
BMI	30 ± 6	30 ± 6	29 ± 6	NS
Comorbidities		*n* (%)		
Lung disease	16(27%)	14(28%)	2(20%)	NS
Hypertension	27(45%)	24(48%)	3(30%)	NS
Heart failure	0	0	0	NS
Ischemic heart disease	2(3%)	2(4%)	0	NS
Vascular disease	4(6%)	3(6%)	1(10%)	NS
Malignancy	2(3%)	1(2%)	1(10%)	NS
Diabetes mellitus	12(20%)	11(22%)	1(10%)	NS
Neurological disease	2(3%)	2(4%)	0	NS
Psychiatric disease	3(5%)	3(6%)	0	NS
Current smoker	3(5%)	2(4%)	1(10%)	NS
Kidney parameters		Mean ± SD or median (IQR) or *n* (%)		
CKD stage ≥ 3 at baseline	6(10%)	6(12%)	0	NS
Baseline CKD stage	2(1–2)	2(2–2)	1(1–2)	NS
Baseline creatinine	77 ± 24	79 ± 25	68 ± 18	NS
Baseline eGFR	78 ± 13	76 ± 13	83 ± 11	NS
Chronic dialysis at baseline	0	0	0	NS
Discharge creatinine	94 ± 84	81 ± 54	162 ± 154	NS
Discharge eGFR	75 ± 23	76 ± 20	65 ± 35	NS
Drugs at admission	*n* (%)			
Steroid treatment	5(8%)	5(10%)	0	NS
ACEi/ARB	20(33%)	19(38%)	1(10%)	NS
Anticoagulants	6(10%)	6(12%)	0	NS
ICU characteristics		*n* (%) or mean ± SD		
SAPS3 at arrival	51 ± 8	52 ± 8	49 ± 11	NS
ICU Length of stay (days)	12 ± 9	12 ± 9	13 ± 12	NS
Lowest PO_2_/FiO_2_ (kPa)	13 ± 6	12 ± 5	15 ± 12	NS
Invasive ventilation (y/n)	27(45%)	23(46%)	4(40%)	NS
Ventilator free days	24 ± 7	25 ± 7	24 ± 10	NS
Mild ARDS	1(2%)	1(2%)	0	NS
Moderate ARDS	2(3%)	2(4%)	0	NS
Severe ARDS	28(47%)	23(46)	5(50%)	NS
Vasopressor (y/n)	36(60%)	29(58%)	7(70%)	NS
Vasopressor free days	26 ± 6	26 ± 6	25 ± 5	NS
AKI (y/n)	33(55%)	26(52%)	7(70%)	NS
Severe AKI (stage 3)	9(15%)	5(10%)	4(40%)	0.016
Acute kidney disease (y/n)	14(23%)	5(10%)	9(90%)	0.019
Dialysis in ICU (y/n)	6(10%)	3(6%)	3(30%)	0.02
Dialysis free days	29 ± 3	29 ± 3	28 ± 3	NS
Chronic dialysis at follow-up	1(1.7%)	0	1(10%)	NS

*ACEi* angiotensin converting enzyme inhibitor, *AKI* acute kidney injury, *ARB* angiotensin II receptor blocker, *ARDS* acute respiratory distress syndrome, *BMI* Body mass index, *CKD* chronic kidney disease, *eGFR* estimated GFR, *ICU* intensive care unit, *IQR* interquartile range, *SAPS3* simplified acute physiology score-3, *SD* standard deviation

Average follow-up time was 18 ± 3 weeks after discharge (range 10 to 26 weeks). Ten patients progressed to a higher CKD stage at follow-up than before contracting COVID-19. Patients with KDIGO stage 3 were more likely to progress to a higher CKD stage (Odds ratio 4.9[1.4–31], *p* = 0.009). Acute kidney disease (AKD) defined as AKI lasting for more than 7 days was associated with progression of CKD (Odds ratio 3.9[1.2–21], *p* = 0.018). There were no other statistical differences between patients who showed CKD progression compared to those that did not with regards to basic demographics, comorbid disease or ICU admission characteristics (Table 1). It should be noted that sarcopenia after critical care would tend to lower creatinine and may lead to over estimation of GFR, and therefore the number of patients with CKD progression may be higher than estimated in this dataset.

The main strength of this study is its comprehensive characterisation of clinical parameters during intensive care as well as comorbid disease. The main limitations of the present dataset are the use of a CKD definition at baseline that depends on creatinine only, a relatively low number of patients leading to few endpoints, and loss to follow-up of 25% of patients without convalescent creatinine.

In conclusion, critical COVID-19 goes with progression of chronic kidney disease in a substantial number of patients. Progression in CKD grade was associated with AKD defined as more than 7 days of AKI, and with KDIGO stage 3 AKI during intensive care.

## Data Availability

The datasets in the current study is available from the corresponding author on reasonable request.
